# Bandgap of Epitaxial Single-Crystal BiFe_1−x_Mn_x_O_3_ Films Grown Directly on SrTiO_3_/Si(001)

**DOI:** 10.3390/ma18092022

**Published:** 2025-04-29

**Authors:** Samuel R. Cantrell, John T. Miracle, Ryan J. Cottier, Skyler Lindsey, Nikoleta Theodoropoulou

**Affiliations:** 1 Materials Science Engineering and Commercialization Program, Texas State University, San Marcos, TX 78666, USA; src105@txstate.edu (S.R.C.); miracle@txstate.edu (J.T.M.); 2 Department of Physics, Texas State University, San Marcos, TX 78666, USA; rjcottier@hrl.com (R.J.C.); skyel@udel.edu (S.L.)

**Keywords:** molecular beam epitaxy, oxide perovskites, bismuth ferrite

## Abstract

We report the growth and optical characterization of single-crystal BiFe_1−x_Mn_x_O_3_ thin films directly on SrTiO_3_/Si(001) substrates using molecular beam epitaxy. X-ray diffraction confirmed epitaxial growth, film crystallinity, and sharp interface quality. Scanning electron microscopy and energy dispersive X-ray spectroscopy verified uniform film morphology and successful Mn incorporation. Spectroscopic ellipsometry revealed a systematic bandgap reduction with increasing Mn concentration, from 2.7 eV in BiFeO_3_ to 2.58 eV in BiFe_0.74_Mn_0.26_O_3_, consistent with previous reports on Mn-doped BiFeO_3_. These findings highlight the potential of BiFe_1_₋_x_Mn_x_O_3_ films for bandgap engineering, advancing their integration into silicon-compatible multifunctional optoelectronic and photovoltaic applications.

## 1. Introduction

As conventional computing architectures struggle to keep pace with the exponential growth in computational demand and the increasing training costs in artificial intelligence (AI), alternative approaches are being explored to integrate data storage and processing within a single platform, thereby enhancing overall efficiency significantly [1]. A particularly promising strategy involves incorporating materials with functional properties absent in silicon alone, such as ferroelectricity, magnetism, or multiferroicity [2]. These properties enable the development of multifunctional devices and in-memory computing applications that overcome the limitations of conventional architectures, offering improvements in both energy efficiency and computing speed [3]. Moreover, the coupling of ferroic, piezoelectric, and optical responses in multiferroics positions them as strong candidates for applications in photovoltaics, photonics, and thin-film acousto-optic devices [4,5].

The integration of SrTiO_3_-on-Si is particularly advantageous, as it provides a direct pathway for incorporating these functional elements into standard complementary metal-oxide-semiconductor (CMOS) technology. Additionally, SrTiO_3_-on-Si serves as a virtual substrate for the epitaxial growth of other perovskite oxides with high crystalline quality, enabling multifunctional devices that leverage spintronics or polarization-based mechanisms for emerging computing paradigms [6].

SrTiO_3_ (STO) is one of the most extensively studied perovskite oxides in both bulk and thin-film forms. It crystallizes in a cubic lattice at room temperature and is commonly used as a substrate for the epitaxial growth of other perovskite oxides. The direct growth of STO on Si was originally proposed as an alternative gate dielectric, owing to its exceptionally high dielectric constant [7]. In 1998, McKee and co-workers successfully achieved the epitaxial growth of single-crystal perovskite STO on Si(001) by depositing a sub-monolayer of Sr at 600 °C on a clean Si(001) surface [8]. Coherent growth of STO (a = 3.905 Å) on Si(001) substrates (a = 5.431 Å) can be achieved using oxide molecular beam epitaxy (MBE) by rotating the STO lattice 45° around the surface normal, aligning STO[001] with Si[011] [9]. Epitaxial STO films grown on Si(001) substrates by MBE are initially commensurately strained to the Si(001) lattice [10]. STO remains coherently strained to Si until a critical thickness t_c_ ≅ 2.4 nm [11,12], beyond which strain relaxation begins. The films are reported to be fully relaxed at thicknesses exceeding 12 nm [13], although the degree of strain relaxation is strongly dependent on the specifics of the growth conditions [14].

Among multiferroic materials, BiFeO_3_ (BFO) has been extensively investigated for applications in electrically controlled spintronic devices as well as ferroelectric and piezoelectric systems [15]. Additionally, BFO diode structures exhibit a substantial visible-light photovoltaic effect. [16] The optical bandgap of BFO is reported to vary between 2.5 and 2.8 eV [17], depending on its form (single-crystal, polycrystalline, or thin films) [18], while BFO grown on STO substrates via oxide MBE has been reported to have a direct bandgap of 2.7 eV [19].

However, BFO, a room-temperature multiferroic material [20], is an antiferromagnet with weak residual magnetism due to a canted spin structure [21,22,23]. In contrast, BiMnO_3_ (BMO) is a ferromagnetic multiferroic material with a Curie temperature of approximately 100 K and ferroelectric transition temperature of 450 K [24]. BMO crystallizes in a monoclinic unit cell, adopting a highly distorted perovskite structure with a pseudo-cubic lattice parameter of 0.395 nm [25,26]. Consequently, BMO thin films grown on STO substrates experience a compressive in-plane strain of 1.1%. Given the structural similarity between BMO and BFO, Mn atoms are expected to readily substitute for Fe atoms, potentially achieving high substitution levels. Additionally, the relatively small lattice mismatch between BMO and STO may facilitate the stabilization of a metastable perovskite phase through strain engineering, analogous to other metastable perovskite systems.

The growth of epitaxial single-crystal BMO and BiFe_1−x_Mn_x_O_3_ (BFMO) thin films on STO(001) substrates has been demonstrated using an adsorption-controlled technique via oxide MBE [27,28]. Furthermore, epitaxial BMO films grown on STO substrates have been reported to exhibit a direct bandgap of 1.1 eV [27]. As Mn substitutes for Fe in BFO, the bandgap was reported to decrease from 2.74 eV, depending on the Mn concentration [28].

We have previously reported growth of BFO thin films on epitaxial STO films deposited on Si substrates [29]. In this work, we incorporate Mn into BFO on epitaxial STO films deposited on Si substrates at doping levels up to 26% using oxide MBE. We investigate the crystallinity and bandgap evolution of epitaxial BFMO thin films grown on STO/Si substrates.

X-ray diffraction (XRD) θ/2θ scans and φ-scans reveal that BFMO films exhibit a single crystallographic orientation, aligned with the Si(001) direction. This confirms out-of-plane epitaxy and a well-defined in-plane orientation with a 45° rotation relative to Si(001). Furthermore, we demonstrate that the films are epitaxial and that Mn substitution in BFO systematically reduces the bandgap, highlighting its potential for optical device applications integrated with Si.

## 2. Materials and Methods

STO films were epitaxially grown on Si(001) using oxide MBE with co-deposition of elemental Sr and Ti sources, and molecular oxygen. The fluxes were adjusted to achieve stoichiometric STO. In situ reflection high-energy electron diffraction (RHEED) was used to continuously monitor the surface quality and stoichiometry during growth. The MBE chamber was operated at a base pressure of ~10^−10^ Torr, with oxygen partial pressures of 2 × 10^−8^ Torr. The substrate temperature was set to 500 °C during STO growth. A detailed description of the STO growth procedure on Si, including Si de-oxidation, is provided in Ref. [30]. This growth process effectively suppresses the formation of an amorphous SiO_2_ layer and promotes two-dimensional growth. Careful oxygen control during nucleation has been demonstrated to facilitate commensurate growth of STO on Si. To maintain a pristine interface and prevent diffusion of oxygen, the STO films were not annealed post-deposition. As we have shown in Ref. [12], under these growth conditions, the interface between Si and STO is atomically and chemically sharp and free of oxide transition layers.

The STO-on-Si (STO/Si) structure serves as a virtual substrate for the deposition of BFO and Mn-substituted BFO, BFMO, which cannot be directly grown on Si. We have previously demonstrated the growth of BFO on 3-inch STO/Si wafers as a virtual substrate [29] using an adsorption-controlled MBE technique. In this process, BFO was deposited using an Fe source and an RF-activated oxygen plasma, with a Bi flux supplied in overpressure to compensate for Bi volatility. The growth rate was controlled by the incoming Fe flux. The oxygen plasma source was generated using an RF plasma source at an oxygen pressure of approximately 10^−6^ Torr. Note that an rf-plasma source (or a mixture of molecular O and ozone) is required for the growth of BFO because of the low reactivity of molecular oxygen at the lower substrate temperatures required for the growth of single-phase films. This adsorption-controlled MBE technique typically used in the case of compound semiconductors has also been previously used for the growth of BFO [19] and BMO [27] on single-crystal STO(001).

Here, we use the adsorption-controlled MBE approach for the growth of BFMO thin films on STO on Si, where the elemental fluxes of Fe and Mn were independently controlled to achieve the desired composition. To ensure complete oxidation of BFMO, an RF oxygen plasma source (300 W) was also used at an oxygen pressure of 10^−6^ Torr. Using these oxygen conditions, BFMO was deposited using Fe, Mn, and oxygen plasma with an overpressure of Bi flux, while the growth rate was controlled by the incoming Fe and Mn flux. Since the standard free energy of oxide formation is greater for Mn than for Fe at all temperatures, decreasing the oxygen pressure enabled a reduction in the substrate temperature. We observed that slowing the growth rate improved film quality. The substrate temperature was maintained at 450 °C and the exact growth parameters were optimized using in situ RHEED. Mn incorporation was controlled by adjusting the Mn cell temperature and verified via using a quartz-crystal monitor (QCM). Films grown under higher substrate temperatures or lower oxygen partial pressures resulted in iron-rich films and the formation of secondary Fe oxide phases, whereas lower substrate temperatures or higher oxygen partial pressures led to bismuth-rich films or bismuth oxides (see Figures S1 and S2). The target Mn compositions ranged from 0 to 30% (x < 0.3). The growth conditions for all Mn-substituted BFO films were the same other than the flux ratio of Fe to Mn. Notably, although initially there is no Si oxide at the interface of STO with Si, the use of oxygen plasma during BFMO growth leads to diffusion of oxygen through STO and the formation of an amorphous SiO_2_ layer at the STO/Si interface [29]. Nevertheless, since the oxygen diffusion is post-deposition of STO on Si, the epitaxial orientation of STO with respect to Si persists. The growth of BFO and BFMO is then initiated on the epitaxial STO layer independent of the interfacial layer between STO and Si.

XRD and X-Ray reflectivity (XRR) measurements were performed using a Rigaku (Tokyo, Japan) SmartLab X-ray diffractometer equipped with a 2 kW sealed tube Cu Kα source. XRR was used to determine the thickness of the BFMO, STO, and SiO_2_ layers along with the associated interface roughness. The XRD measurements were conducted in a high-resolution parallel beam configuration. θ/2θ and φ-scans scans (wide-angle, substrate aligned) were acquired with a Ge(220 × 2) monochromator on the incident beam side. For all scans, narrow (1.00 mm) vertical incident (divergence) and receiving (scattering) slits were used to reduce the angular beam divergence into the diffraction plane. A narrow (2 mm) horizontal incident slit and a 5° receiving Soller slit were used to reduce the axial beam divergence perpendicular to the diffraction plane, thus reducing the asymmetry of the diffraction peaks and improving the accuracy of the Bragg angle position. The resolution of the diffracted beam in the high-resolution scans was less than 0.01°. The XRR measurements were analyzed using Rigaku’s GlobalFit ver. 2.1.1.0 software, which models and fits the reflectivity curves by iteratively optimizing parameters such as layer thickness, surface roughness, and electron density.

Film morphology was examined via scanning electron microcopy (SEM), while Mn incorporation was quantitatively assessed using energy dispersive X-ray spectroscopy (EDS) with an accelerating voltage of 5 kV and current of 2.7 nA. Measurements were conducted using an Thermo Fisher Scientific (Waltham, MA, USA) Helios NanoLab 400 DualBeam system (Thermo Fisher Scientific, https://www.thermofisher.com/us/en/home/brands/thermo-scientific.html, accessed on 24 April 2025) equipped with an EDAX module and Thermo Fisher Scientific Axia ChemiSEM, achieving a Mn concentration accuracy within a few percent. The optical properties and bandgap were determined via spectroscopic ellipsometry using a J.A. Woolam M-2000 spectroscopic ellipsometer (J. A. Woolam, Lincoln, NE, USA). Data analysis and modeling were performed using the J. A. Woolam CompleteEASE ver. 6.54 software package from the same manufacturer. Ellipsometry spectra (Ψ,Δ) were collected at room temperature over a 0.7 to 4 eV spectral range, with a 20 meV step size, at four angles of incidence (55°, 60°, 65°, and 70°) which have been previously used and shown to work well for these types of films.

## 3. Results and Discussion

### 3.1. X-Ray Diffraction (XRD)

**Figure 1:**
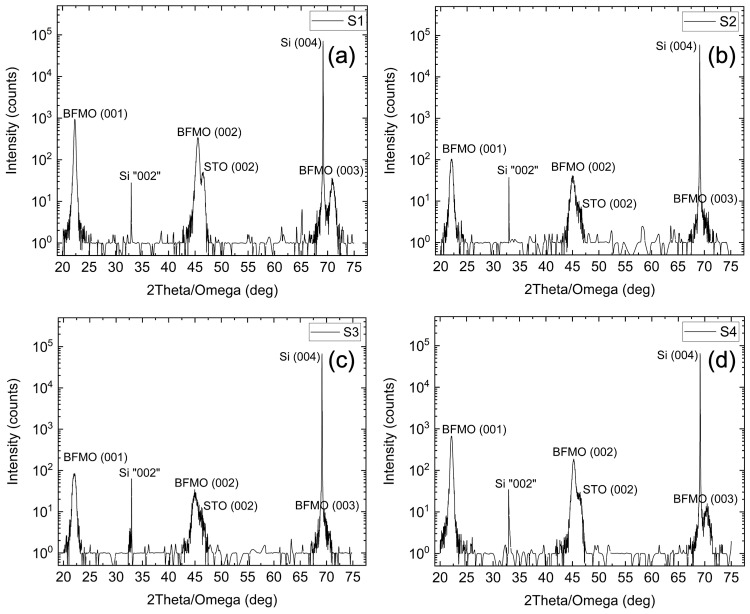
θ/2θ XRD scans of all samples: (**a**) S1, (**b**) S2, (**c**) S3, and (**d**) S4. They all show single-crystal film and no evidence of second phases.

The crystalline quality of the films was assessed using XRD. Figure 1 shows high- resolution survey scans (θ/2θ) for samples (a) S1, (b) S2, (c) S3, and (d) S4. All peaks can be ascribed to Si(00l), STO(00l) or BFMO(00l) (l = 1, 2, 4), showing epitaxy along the c-axis. Only (00l) peaks are observed, indicating a single crystallographic orientation with the out-of-plane epitaxial relationship BFMO (001)||STO(001)||Si(001) [31].

The BFMO(001) and STO(001) peaks overlap for all samples since their angular separation is smaller for lower angles. As the diffraction angle increases, the BFMO(002) and STO(002) peaks start separating and at higher angles, the BFMO(004), STO(004) peaks are distinct as their spectral width is smaller than their angular separation. No secondary phases are detected, denoting that the positions of the BFMO films are single phase and oriented along the c-axis. The c-lattice constants from the BFMO(002) peaks are 3.977 Å, 4.016 Å, 4.013 Å, and 3.991 Å for S1, S2, S3, and S4, respectively, which is consistent with the lattice constant for the rhombohedral crystal structure for BFO. The BFO film (S1) with a thickness of 50 nm is fully relaxed. For S2, S3, and S4, the c-lattice constants correspond to fully relaxed BFMO as expected. The lattice constants and residual strain, of course, depend not only on thickness but on the exact growth procedure, the most important of which is the oxygen pressure.

**Figure 2:**
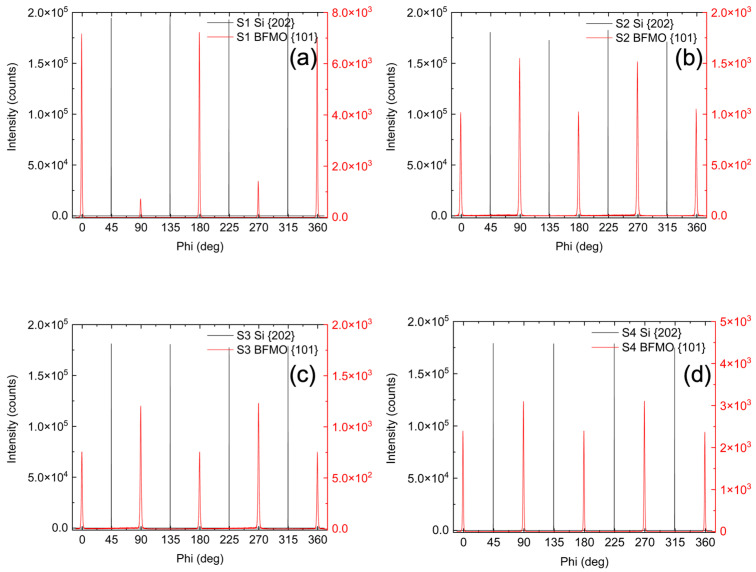
φ-scans of all samples, (**a**) S1, (**b**) S2, (**c**) S3, and (**d**) S4, around the BFMO{101} diffraction peaks, overlaid with φ-scans of the Si substrate around the {202} diffraction peaks, revealing a fourfold symmetry with the expected 45° in-plane rotation relative to the surface normal.

To determine the in-plane orientation of the films relative to the substrate, φ (azimuthal) scans were performed around the {103} diffraction peaks of the BFMO films [Figure 2, red lines]. The φ-scans exhibit a clear four-fold 90° rotational symmetry [Figure 2, red lines] in the {103} planes relative to the surface normal, indicating that the films possess a well-defined in-plane orientation. The φ-scans around the {202} diffraction peaks of the Si substrate overlaid on the same plot [Figure 2, black lines] also show a four-fold symmetry with the expected 45° rotation around the surface normal. These scans are held at a constant inclination away from the surface normal and the persistent signal indicates that each of these planes maintain nearly the same angle with respect to the primary out of plane crystal axis; however, the angular resolution of the φ-scans is insufficient to detect minute angular deviations. The identical rotational dependencies and four-fold symmetry observed in the φ-scans for all films in Figure 2 further confirm that the BFMO films are single-crystalline and maintain good epitaxy with the original Si substrate even after formation of the amorphous SiO_2_ layer from the oxygen plasma as previously described. The films are aligned with the expected 45° rotation with respect to Si(100) around the surface normal, consistent with the epitaxial relationship BFMO [100]//STO [100]//Si [110].

Together, the XRD data show a single phase of BFMO coherent with the substrate but rotated by 45° about the Si(004) axis with respect to the Si substrate. The φ-scans show four-fold symmetry in-plane, indicating a pseudo-cubic structure, and the calculated lattice constants further indicate that BFMO is rhombohedral and that the films are fully relaxed.

### 3.2. X-Ray Reflectivity (XRR)

**Figure 3:**
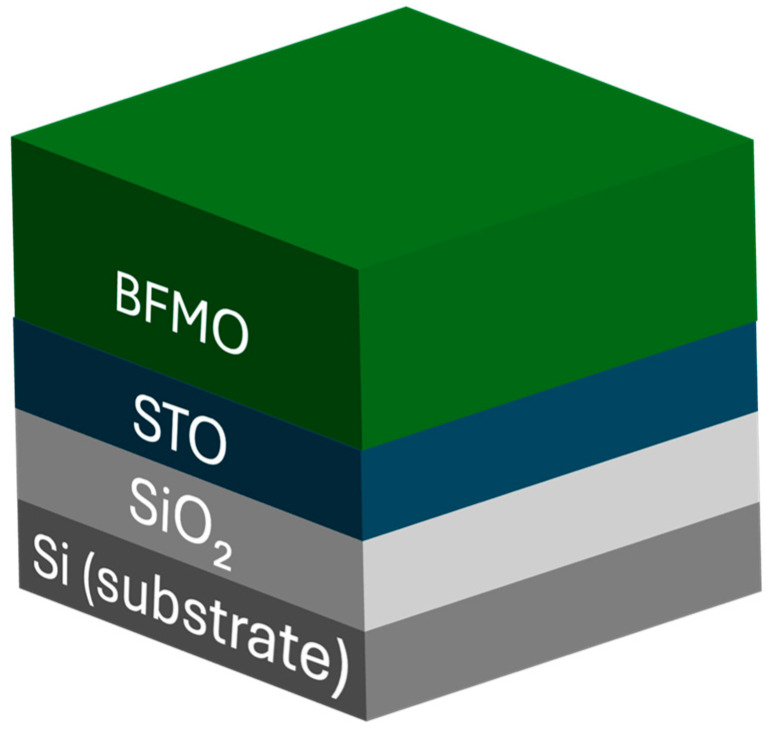
Schematic of the film stack used for XRR and ellipsometry analysis.

XRR measurements were performed to determine the thickness and roughness of each of the identified layers and interfaces, the structure of which is subsequently shown in Figure 3.

**Figure 4:**
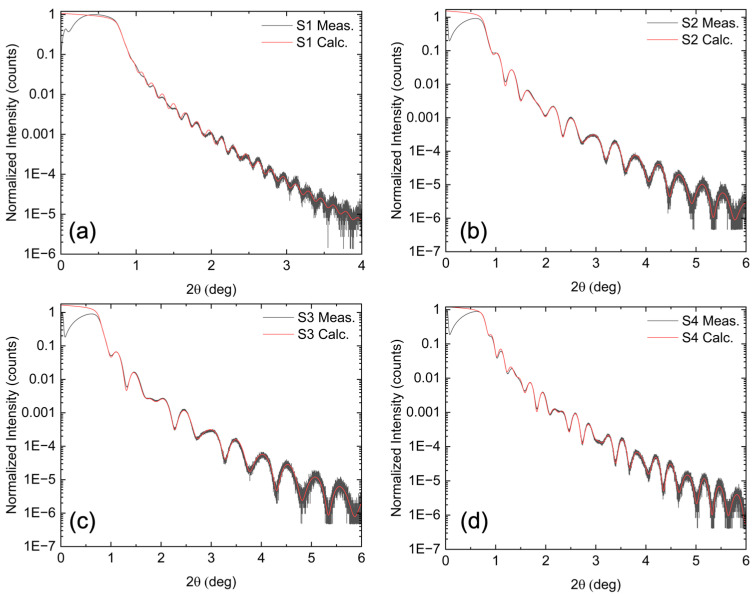
XRR experimental data (black line) and fittings (red line), using the model shown in Figure 3, of all samples: (**a**) S1, (**b**) S2, (**c**) S3, and (**d**) S4.

Figure 4 shows the direct XRR measurements (black lines) for all samples. With the exception of S1, clear oscillations are observed up to 6°, indicating sharp interfaces and a smooth surface. These data were used to calculate a model fit (red lines) using the model in Figure 3, which includes the Si substrate, Si oxide, STO film, and final BFMO film. Allowed fitting parameters included the thickness and roughness of each of these layers. As shown in Figure 4, the model closely reproduces the experimental data. The relaxed parameters from the fitting results along with their standard deviations are shown in Table 1, where uncertainty is on the order of the last decimal place.

The STO layer, used as a virtual substrate for BFMO growth, varied in thickness between 7.8 and 10 nm for the Mn-substituted films (S2, S3, S4), while it was 16 nm for the pure BFO film (S1). In all cases, the STO thickness exceeded the 2.4 nm critical thickness for STO on Si. The BFMO layer thicknesses were 20 nm (S2), 17 nm (S3), and 27 nm (S4), while the BFO layer in S1 was significantly thicker at 49 nm. This increased thickness in S1 also led to a thicker SiO₂ interfacial layer due to the longer growth time. The interface roughness between STO and BFMO was less than half a monolayer, and the BFMO surface roughness remained near the atomic level.

Although the BFO and Mn-substituted BFMO films have different thicknesses, this should not significantly impact the calculated bandgap. Prior studies have reported that the optical bandgap of epitaxial BFO thin films is independent of thickness or the out-of-plane lattice constant [18]. Nevertheless, precise thickness determination via XRR is crucial, as these results serve as seed values for the subsequent ellipsometry analysis.

### 3.3. Energy Dispersive X-Ray Spectroscopy (EDS)

EDS analysis was performed to determine the incorporated Mn concentration within the BFMO film of each sample. The values in Table 2 represent the average and standard deviation as measured across five separate locations around the most uniform center area of each sample, as detailed in the Supplementary Materials. As expected, no significant Mn was detected in the BFO of S1, while the BFMO of S2, S3, and S4 was verified to contain 4%, 12%, and 26% Mn incorporation, respectively.

### 3.4. Ellipsometry

Due to the narrow thickness of these films, the ellipsometry spectra collected from these samples include contributions from all layers. The optical properties for the Si, SiO_2_, and STO layers were obtained from the CompleteEASE analysis suite’s standard material files and verified against previously grown STO on Si samples.

As with XRR, the sample stack structure shown in Figure 3 was used as a base and interfacial layers were incorporated to account for interlayer roughness. Specifically, an interface layer is added between the SiO₂ layer and the Si substrate, along with an effective medium approximation (EMA) intermediate layer between the SiO₂ and STO films to properly model interface roughness. The fitted ellipsometry layer thicknesses were constrained to within 10% of the values previously determined via XRR. The relaxed fitting parameters from these models are presented in Table 3, where uncertainty is on the order of the last decimal place.

**Figure 5:**
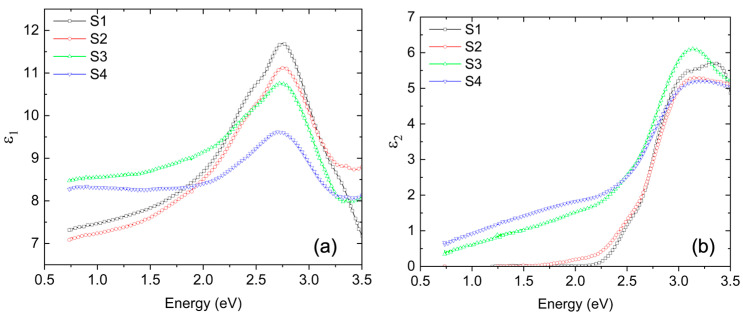
(**a**) Real and (**b**) imaginary parts of the calculated dielectric function of BFMO layers for all samples.

A wavelength-by-wavelength approach was used to model the BFMO top layer as the only unknown material within the stack. Since no physical line-shape model was implemented, this method allows for an unbiased extraction of the intrinsic dielectric function. The approach yielded realistic optical properties for the BFMO layers, with a final mean squared error less than 3 for all samples. Table 2 details the extracted values for thickness and roughness for all films. The extracted components of the complex dielectric function are shown in Figure 5, displaying the calculated (a) ε1 (real part) and (b) ε2 (imaginary part) for all samples.

**Figure 6:**
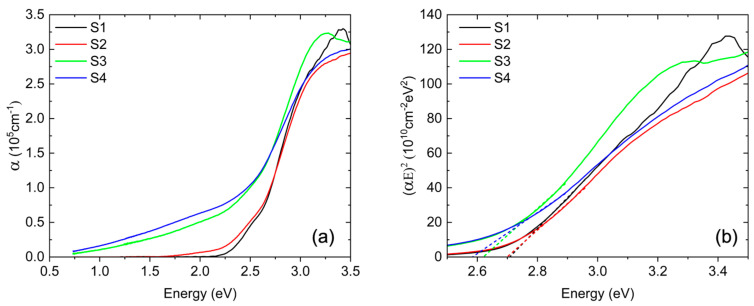
(**a**) Calculated absorption coefficient and (**b**) Tauc plots of the BFMO layers for all samples. The dashed lines represent the linear fits to the Tauc plots.

Figure 6a shows the experimental energy dependence of the BFMO absorption coefficient extracted from spectroscopic ellipsometry data. The absorption coefficients for S1 and S2 exhibit a similar trend, with the onset of optical absorption for S2 occurring at a slightly lower energy than in S1. As the Mn concentration increases, the absorption onset decreases in energy, and for S3 and S4, it is much lower, starting at ~1 eV. Previous ellipsometry studies for BFMO films with 50% Mn grown on STO(001) have shown high absorption in the infrared, which is similar to our results [32]. Additionally, the absorption spectra of all samples exhibit a distinct feature centered around 3.2 eV, which has been previously attributed to a higher-energy charge-transfer transition in BFO, as supported by both theoretical calculations [33] and experimental results [34].

The corresponding Tauc plots, (αE)^2^ vs. E, are presented in Figure 6b. The bandgap values for each sample are extracted from the intercepts of the linear fits in Figure 6b, as shown by the dashed lines for each respective sample. For the undoped BFO in S1, the direct bandgap is determined to be ~2.7 eV, which is in excellent agreement with previous reports on bulk crystals [35] and thin films [33]. However, optical excitations begin at a slightly lower energy with a shoulder near 2.5 eV in α(E), as previously observed [35].

**Figure 7:**
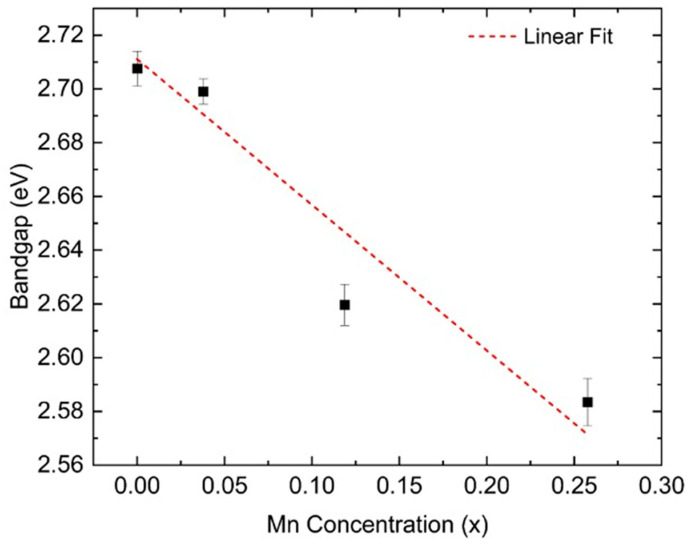
Calculated BFMO bandgap as a function of the Mn concentration.

Figure 7 illustrates the relationship between Mn concentration and the progressive redshift of the bandgap onset in these films. For the sample with 4% Mn substitution (S2), the calculated bandgap is slightly lower than for S1. The bandgap decreases further to approximately 2.62 eV for S3 (12% Mn) and 2.58 eV for S4 (26% Mn). This trend is consistent with previous ellipsometry studies of BFMO films on STO(001) substrates, which have also reported a bandgap reduction with increasing Mn concentration [28]. Furthermore, BiMnO_3_ thin films have been shown to exhibit a bandgap of 1.1 eV [27], supporting the expectation that Mn substitution leads to a systematic decrease in the BFMO bandgap.

## 4. Conclusions

We have demonstrated the epitaxial growth of BFMO thin films on SrTiO_3_-buffered Si(001) using MBE, achieving Mn substitution levels up to 26%. XRD and XRR confirmed high crystalline quality and well-defined interfaces, while SEM and EDS validated the film uniformity and composition. Optical characterization via spectroscopic ellipsometry showed that Mn incorporation progressively lowers the bandgap, with a reduction from 2.7 eV in BiFeO_3_ to 2.58 eV in BFMO with 26% Mn.

Lowering the bandgap is advantageous for optoelectronic and photovoltaic applications, as it allows for increased absorption in the visible and near-infrared range. Notably, the bandgap reduction occurred without significant lattice distortion or secondary phase formation, highlighting the viability of Mn doping for bandgap engineering in perovskite oxides. Future work will focus on exploring Mn concentrations at higher concentration ranges where the most pronounced bandgap shifts are expected and further investigating the electronic and transport properties of BFMO for potential device integration.

## Figures and Tables

**Table 1:** Fitting results from the XRR analysis.

Sample:	SiO_2_ Thickness (nm)	SiO_2_ Roughness(nm)	STO Thickness (nm)	STO Roughness (nm)	BFMO Thickness (nm)	BFMO Roughness (nm)
S1	10.2	2.1	16.2	1.1	49.5	0.6
S2	1.3	1.0	7.8	0.3	20.1	0.4
S3	1.4	1.1	7.7	0.3	16.8	0.4
S4	0.4	0.6	10.2	0.24	26.9	0.4

**Table 2:** Mn concentration determined by EDS.

Sample:	EDS Mn x
S1	0.0 ± 0.003
S2	0.04 ± 0.01
S3	0.12 ± 0.01
S4	0.26 ± 0.02

**Table 3:** Fitting results from ellipsometry analysis.

Sample:	Interface Thk (nm)	SiO_2_ Thk (nm)	EMA Thk (nm)	EMA Mixing %	STO Thk (nm)	BFMO Thk (nm)	Roughness (nm)	MSE
S1	2.0	6.0	4.0	47	9.4	49.1	3.0	2.96
S2	1.6	1.0	1.0	69	11.0	26.7	1.0	0.60
S3	0.1	1.0	1.2	31	8.0	20.1	1.1	0.45
S4	0.1	2.2	3.8	37	7.3	18.6	1.7	0.42

## Data Availability

The original contributions presented in this study are included in the article/Supplementary Material. Further inquiries can be directed to the corresponding author.

## Supplementary Materials

The following supporting information can be downloaded at: https://www.mdpi.com/article/10.3390/ma18092022/s1, Figures S1–S4: SEM images of all samples; SEM image of a sample with spotty RHEED during growth; EDS spectra of all samples; typical AFM image.

## Abbreviations

The following abbreviations are used in this manuscript:

BFO	BiFeO_3_
BFMO	BiFe_1−x_Mn_x_O_3_
BMO	BiMnO_3_
CMOS	Complementary Metal-Oxide-Semiconductor
EDS	Energy Dispersive X-Ray Spectroscopy
EMA	Effective Medium Approximation
MBE	Molecular Beam Epitaxy
NSF	National Science Foundation
RHEED	Reflection High-Energy Electron Diffraction
SEM	Scanning Electron Microcopy
STO	SrTiO_3_
XRD	X-Ray Diffraction
XRR	X-Ray Reflectivity
