# Supplementary material for: Bandgap of Epitaxial Single-Crystal BiFe1−xMnxO3 Films Grown Directly on SrTiO3/Si(001)
Source: Materials (Basel). 2025 Apr 29;18(9):2022. doi: 10.3390/ma18092022 (PMC12072848; doi:10.3390/ma18092022)
Supplement: Supplementary file 1 [file materials-18-02022-s001.zip › materials-3585064-supplementary.pdf]

materials

MDPI

Article

Bandgap of Epitaxial Single-Crystal $BiFe_{1-x}Mn_xO_3$ Films Grown Directly on $SrTiO_3$/Si(001)

Samuel R. Cantrell [1], John T. Miracle [1], Ryan J. Cottier [2,†], Skyler Lindsey [2] and Nikoleta Theodoropoulou [1,2,*]

1 Materials Science Engineering and Commercialization Program, Texas State University, San Marcos, TX 78666, USA; src105@txstate.edu (S.R.C.); miracle@txstate.edu (J.T.M.)
2 Department of Physics, Texas State University, San Marcos, TX 78666, USA; rjcottier@hrl.com (R.J.C.); skyel@udel.edu (S.L.)
* Correspondence: ntheo@txstate.edu

Supplemental Information

Scanning Electron Microscopy (SEM) and Energy Dispersive X-Ray Spectroscopy (EDS)

SEM imaging, shown in Figure SI1, reveals continuous films without large grains, holes, or other inclusions. In fact, the SEM images appear featureless, as expected for epitaxial films. In contrast, Fig. SI5 shows the SEM image of a BFO sample that showed spotty RHEED patterns during growth. While SEM lacks the sensitivity of XRD for detecting secondary phases, it provides a quick and effective assessment of sample quality and surface morphology after growth. For all samples, SEM images confirm the absence of surface defects, such as large grains, voids, or inclusions which we typically observed in samples grown at conditions away from the ideal.

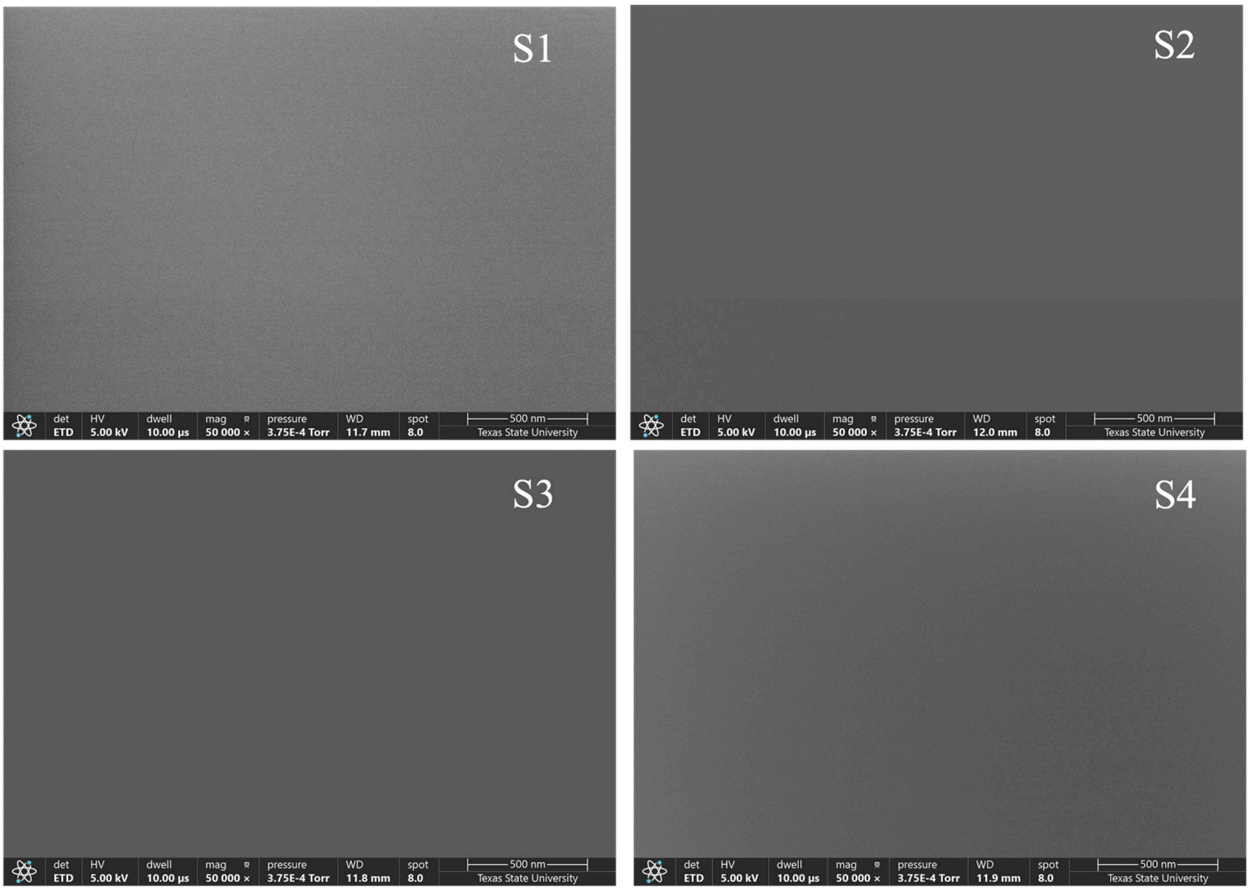

Figure S1: SEM images of S1 (top left), S2 (top right), S3 (bottom left), S4 (bottom right)

The Mn concentration in each sample was determined using EDS with an accelerating voltage of 5 kV and a current of 2.7 nA. The reported values represent the average and standard deviation from measurements taken at five distinct locations near the center of each sample.

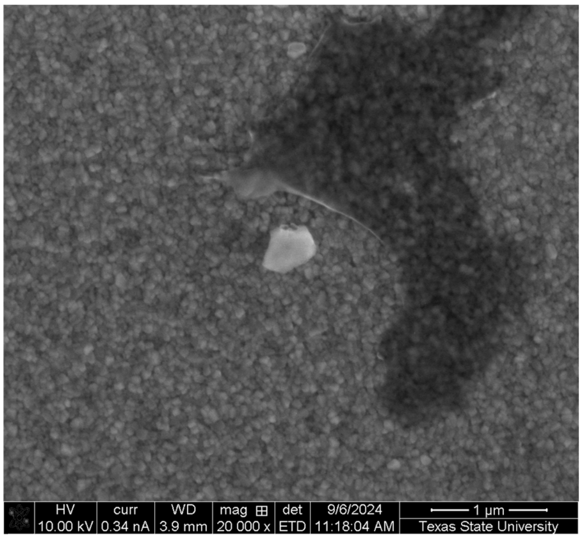

Figure S2: SEM image of a BFO sample grown away from the ideal conditions that showed spotty RHEED during growth.

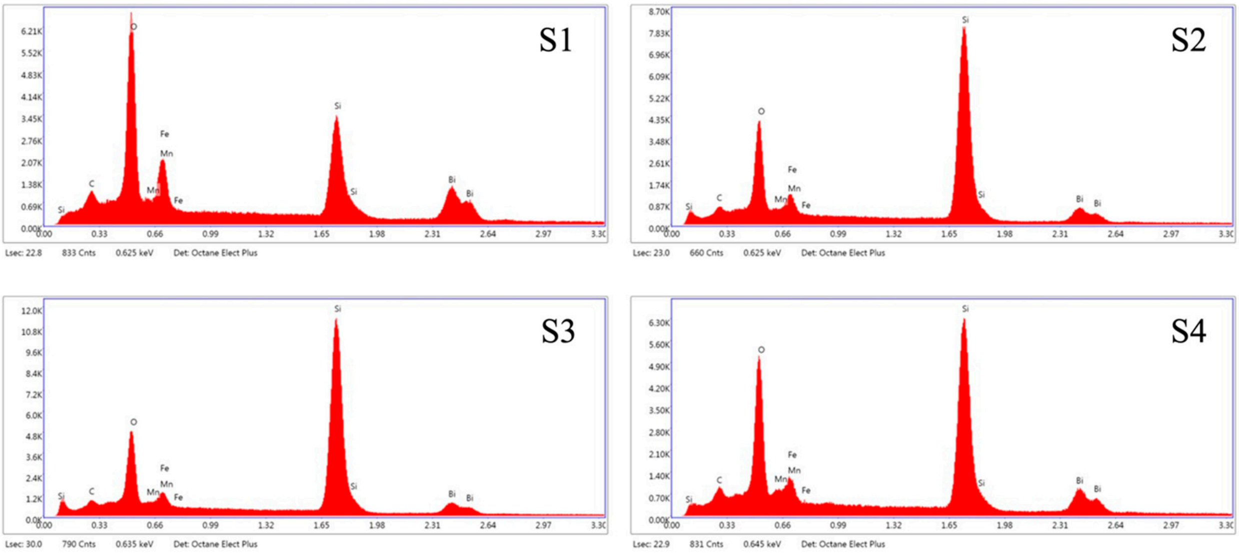

Figure S3: EDS spectra of S1 (top left), S2 (top right), S3 (bottom left), S4 (bottom right)

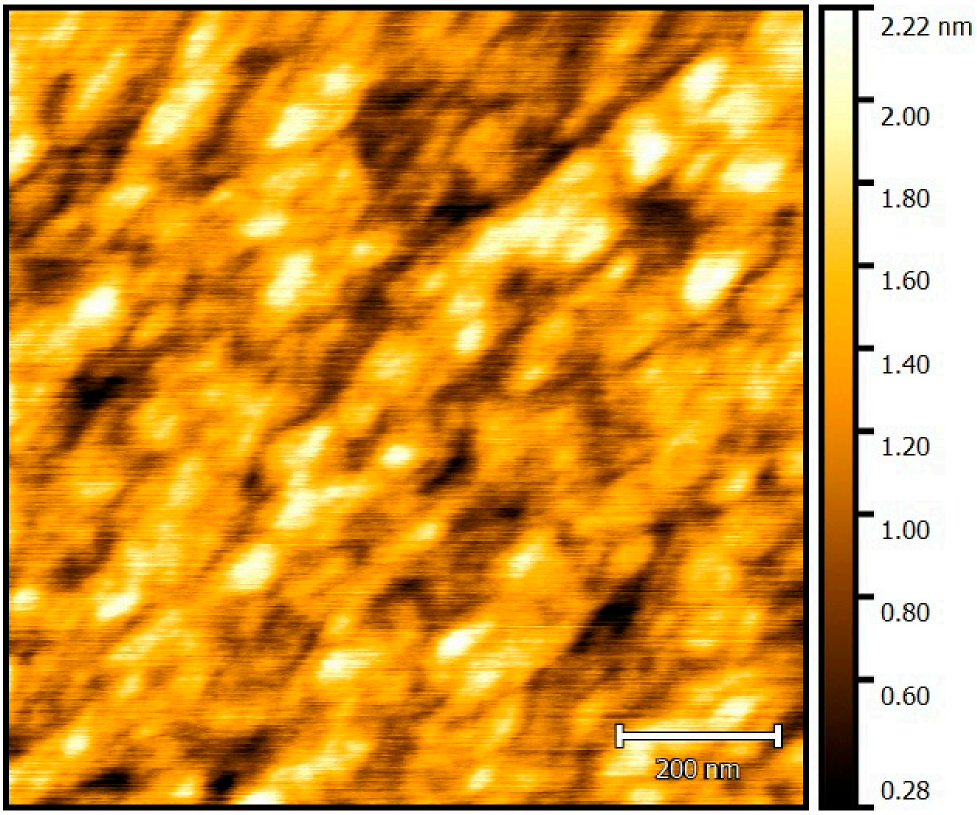

Figure S4: Typical Atomic Force Microscopy image (S1) showing low RMS roughness.
